# Exploring the Diagnostic Challenges of Uterine Lesions in Frozen Section: A Comprehensive Study at a Tertiary Care Center

**DOI:** 10.7759/cureus.58577

**Published:** 2024-04-19

**Authors:** Neelayadakshi B., Sudha V.

**Affiliations:** 1 Department of Pathology, Saveetha Medical College and Hospital, Saveetha Institute of Medical and Technical Sciences, Saveetha University, Chennai, IND

**Keywords:** endometrial polyp, xanthogranulomatous degeneration, hyaline degeneration, leiomyoma, leiomyosarcoma, endometrial carcinoma, carcinosarcoma, accuracy of frozen section, frozen section analysis, uterine masses

## Abstract

Introduction

Uterine masses are commonly submitted for frozen section, to guide the surgeon, regarding the type and extent of the procedure during surgery. Despite the technical difficulties in processing, sectioning, and staining of frozen section samples, it remains a fairly reliable intraoperative tool.

Aim

This study aims to analyze the diverse spectrum of uterine masses sent for frozen sections for two years. In addition, it aims to analyze the histomorphology of the uterine masses sent for the frozen section and correlate it with that of the routine histopathological findings, thereby justifying the diagnostic value of the frozen section with this study. Furthermore, the study aims to classify the lesions into benign and malignant, quantify their frequency, and list the most common lesions seen in the uterine mass specimens sent for frozen section analysis.

Methodology

This retrospective descriptive study includes data from January 2021 to December 2022, retrieved from the archives of the Department of Pathology at Saveetha Medical College. This study includes a total of 76 cases, including all the uterine masses sent for frozen section analysis during the study period.

Results

Of the total of 76 cases received, 17 (22.4%) were malignant and 59 (77.6%) were benign. Of the malignant cases reported, the most common was endometrial carcinoma, and the least common entities encountered were carcinosarcoma and leiomyosarcoma. Of the benign cases, benign endometrial polyp was the most common endometrial lesion and leiomyoma with and without degeneration was the most common myometrial lesion encountered. Of the 50 cases of leiomyoma encountered, 16 had extensive degenerative changes. The most common degeneration seen in the fibroid was hyaline degeneration, and the least common was xanthogranulomatous degeneration.

Conclusions

The intraoperative frozen section analysis is a very important diagnostic tool, but we need to be aware of its limitations. The accuracy, sensitivity, and specificity rates were found to be high. Thus, frozen section diagnoses can be very valuable in the clinical management of uterine tumors. Careful gross examination, sampling from representative areas, and good communication between the pathologist and surgeon may help in avoiding its limitations.

## Introduction

Uterine masses are commonly submitted for frozen section, to guide the surgeon, regarding the type of surgery and the extent of the procedure in the operation theater [1]. The most important use of the frozen section is to determine whether the tissue being sampled is benign or malignant [2]. It may also help in determining the adequacy of a resection margin during surgery [2]. Despite the technical difficulties in processing, sectioning, and staining of frozen section samples, it remains fairly reliable with an accuracy of 91.5% to 97.4% [2]. The purpose of this study is to analyze the diverse spectrum of uterine masses sent for frozen section analysis for two years. In addition, this study aims to analyze the histomorphology of the uterine masses sent for frozen sections and correlate it with that of the routine histopathological findings. Furthermore, this study aims to classify the lesions into benign and malignant, quantify their frequency, list the most common lesions seen in the specimens sent for frozen section analysis, and find the diagnostic value, sensitivity, specificity, and accuracy of the frozen section according to the status of malignancy in this study. This was presented at the 2023 Transmission and Distribution Automated Power Conference (TAPCON) as an oral presentation on July 21, 2023.

## Materials and methods

This study was conducted in the Pathology Department at Saveetha Medical College, for two years, from January 2021 to December 2022. This was a retrospective descriptive study that included all the uterine masses sent for frozen section analysis during those two years. A complete enumeration sampling method was used and a total of 76 cases were included in the study. Fresh tissue from the operation theater was sent in a container without any fixative to the Histopathology Department at Saveetha Medical College, along with the request form. A gross examination of the specimen was done. The specimen was dissected, and sections were taken from representative areas. Frozen sectioning was carried out using a Leica cryostat set at a temperature range of -18 to -26 °C. Four-micrometer-thick sections were taken. The slides were stained with rapid hematoxylin and eosin method. The slides were then reviewed, and the frozen section diagnosis was conveyed to the operating surgeon within 20 minutes.

Following this, the specimen was fixed in 10% neutral buffered formalin for routine histopathological examination (HPE). After the specimen was adequately fixed, grossing was done and sections were taken from the representative areas using standard guidelines. The tissue sections were then processed using an automated tissue processor, and the slides were prepared. Routine hematoxylin and eosin staining was done. The slides were then reviewed, and a histopathological diagnosis was given. The frozen section findings were correlated with the routine histopathological diagnosis. The subsequent statistical analysis was done using SPSS software, and the sensitivity, specificity, and accuracy of frozen section diagnosis were determined. The photos of the histopathological sections and the gross image were taken using a smartphone device.

The lesions were classified into benign and malignant, and their frequency was quantified. The most common uterine mass lesions sent for frozen section analysis were then listed. Descriptive variables were analyzed by frequency and proportion.

## Results

**Figure 1:**
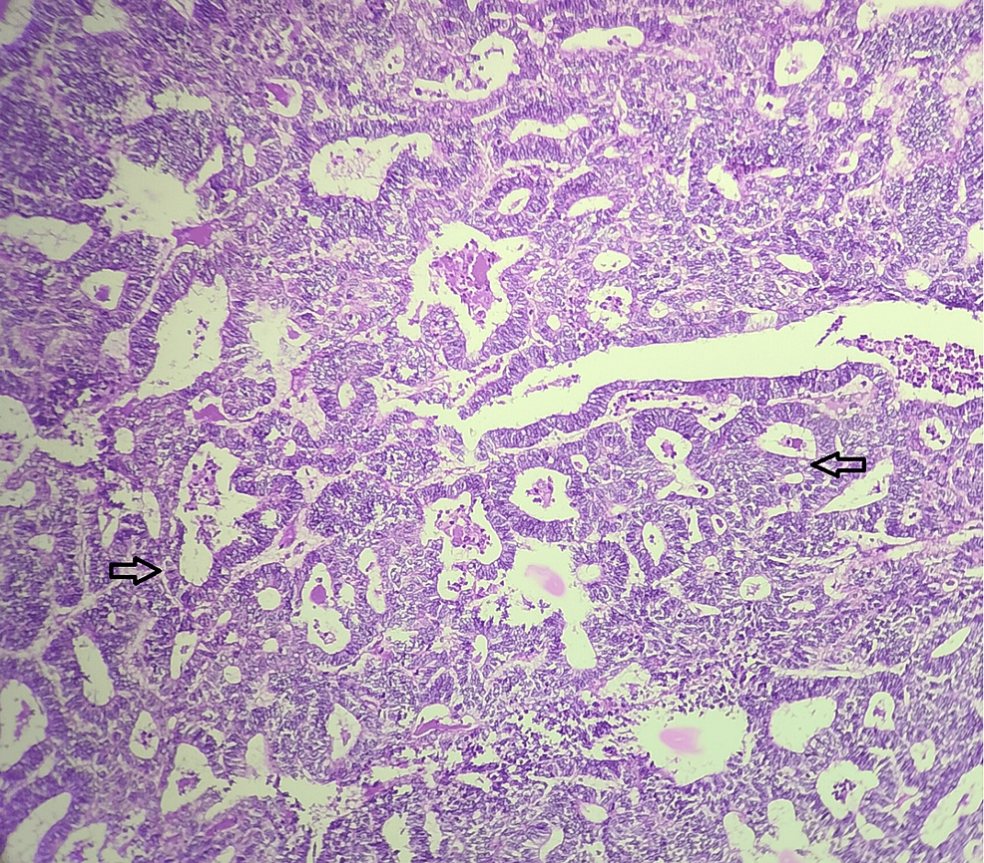
Well-differentiated endometrial carcinoma - x10 magnification; hematoxylin and eosin stain. The section shows a back-to-back arrangement of atypical endometrial glands with irregular contours and minimal intervening stroma. The arrows indicate atypical endometrial glands. Source: Neelayadakshi B.

**Figure 2:**
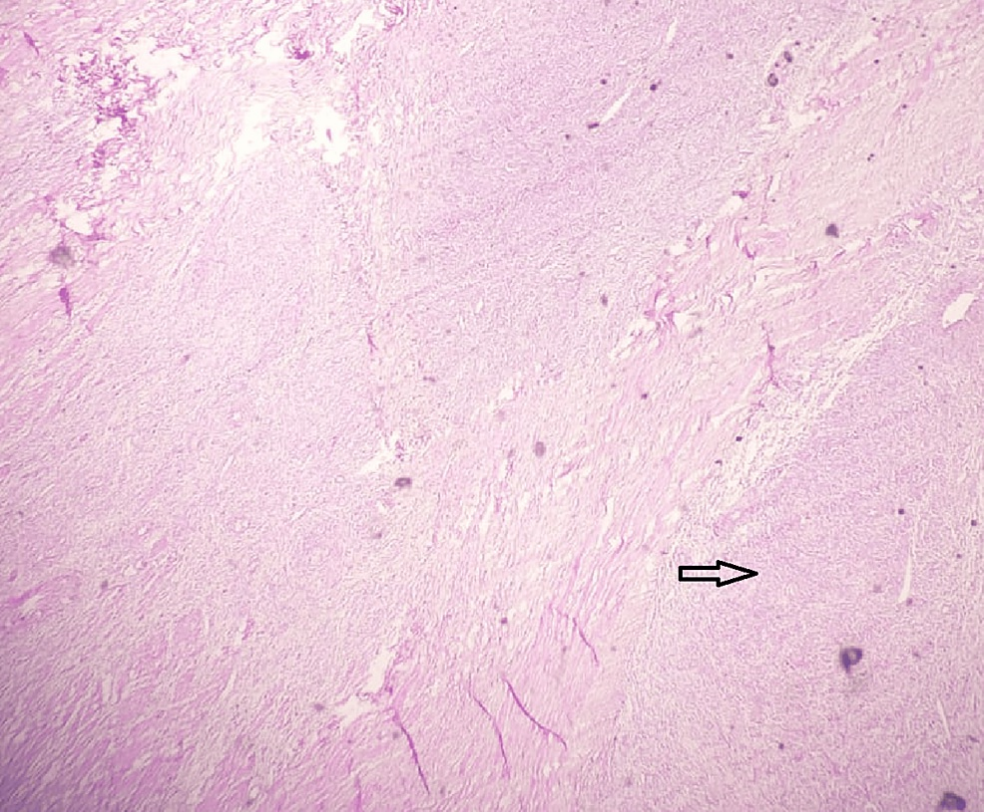
Low-grade endometrial stromal sarcoma; hematoxylin and eosin stain; scanner view. The section shows tongue-like projections of the tumor (indicated by the arrow), infiltrating into the adjacent myometrium. Source: Neelayadakshi B.

A total of 76 cases were analyzed for two years, of which 17 cases (22.4%) were malignant and 59 cases (77.6%) were benign. Of the malignant cases reported, 11 cases (14.4%) were endometrial carcinoma (Figure 1), which was the most common malignant entity encountered. Two cases (2.6%) were endometrial stromal sarcoma (Figure 2).

**Figure 3:**
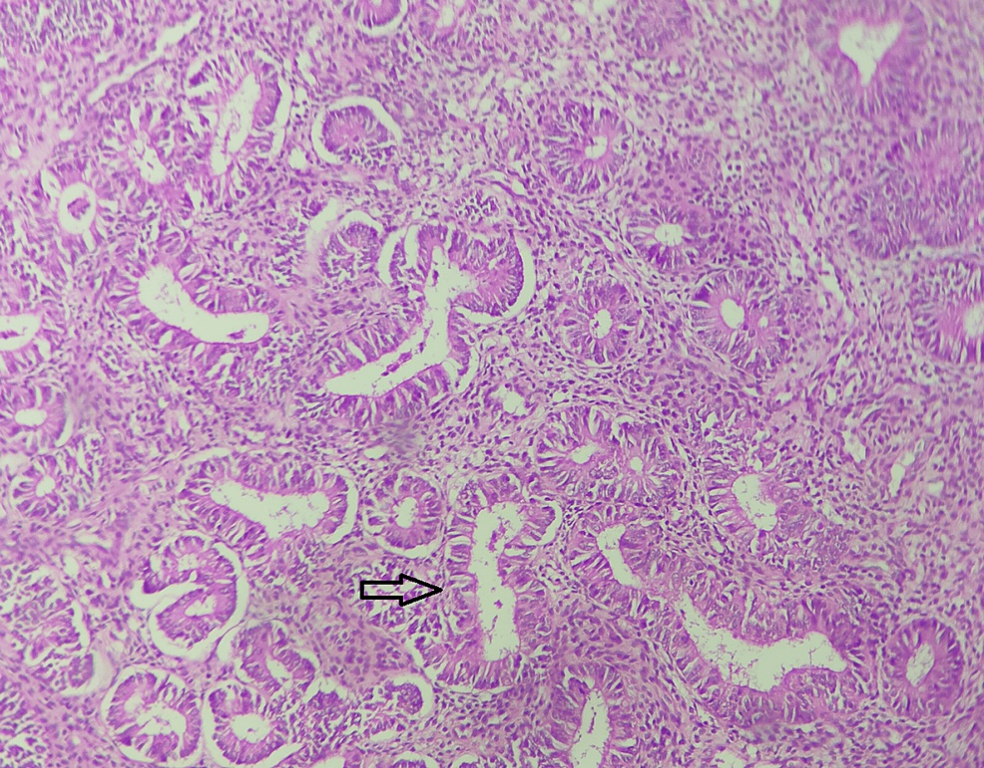
Atypical endometrial hyperplasia; hematoxylin and eosin stain; x10 magnification. The section displays closely packed endometrial glands with an increased gland-to-stromal ratio, but stroma is visible between the glandular basement membranes. Variation in gland size is seen with mild nuclear atypia. The arrow indicates a dilated endometrial gland showing mild nuclear atypia in its lining epithelium. Source: Neelayadakshi B.

Two cases (2.6%) were atypical endometrial hyperplasia (Figure 3).

**Figure 4:**
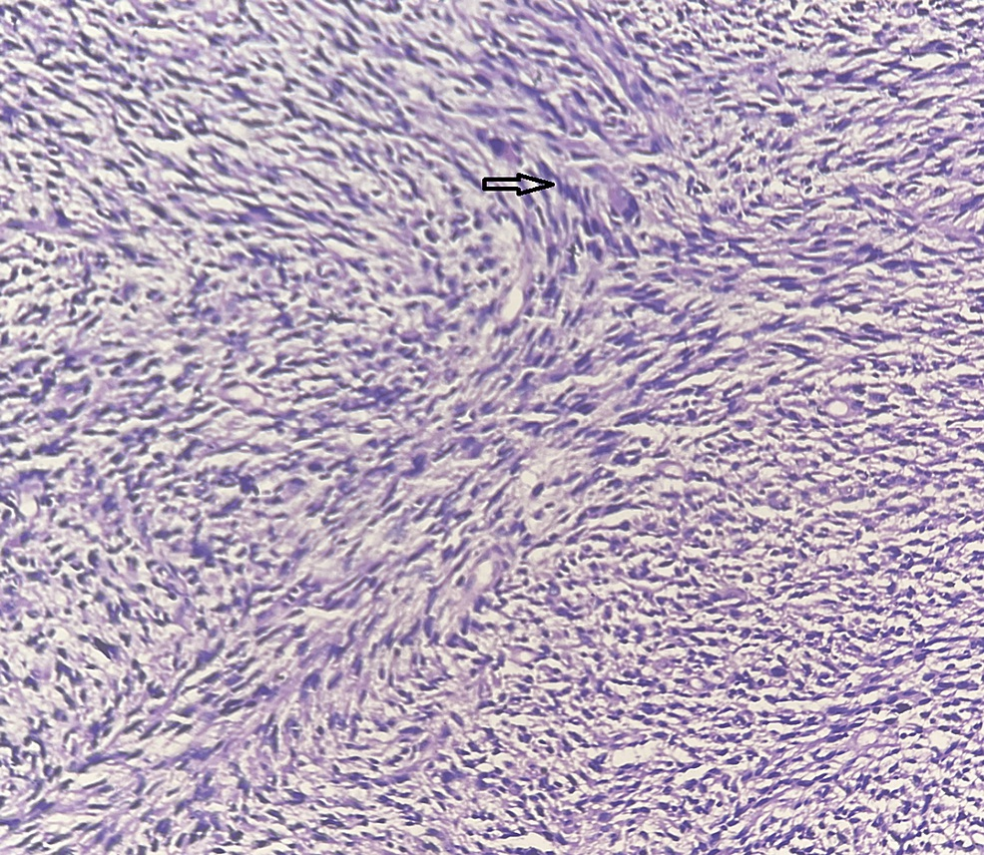
Leiomyosarcoma - x10 magnification; hematoxylin and eosin stain. The section studied shows atypical spindle cells arranged in fascicles, having a moderate amount of cytoplasm, with pleomorphic and hyperchromatic nuclei. The arrow shows the atypical spindle cells with hyperchromatic nuclei. Source: Neelayadakshi B.

One case (1.3%) was leiomyosarcoma (Figure 4).

**Figure 5:**
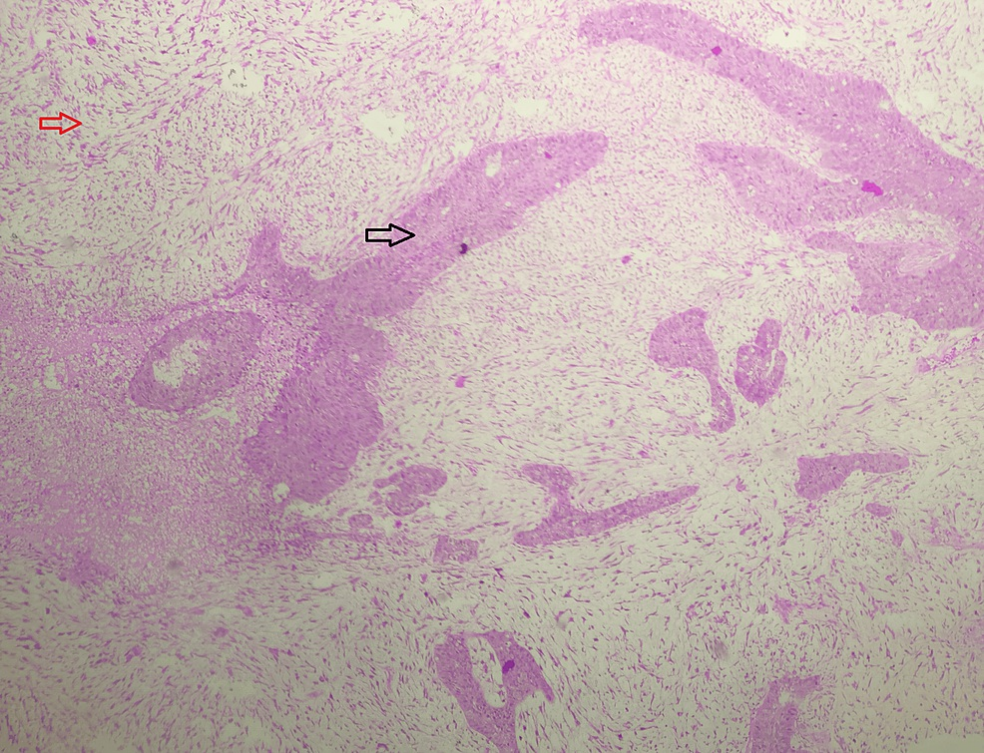
Carcinosarcoma; hematoxylin and eosin stain; scanner view. The section shows a biphasic tumor comprising the malignant epithelial component (indicated by the black arrow) and the malignant mesenchymal component (indicated by the red arrow). Source: Neelayadakshi B.

One case (1.3%) was reported as carcinosarcoma (Figure 5).

Thus, the least common malignant entities encountered were carcinosarcoma and leiomyosarcoma. Of the benign cases reported, benign endometrial polyp (18, 23.6%) was the most common endometrial lesion seen and leiomyoma with and without degeneration (50, 65.6%) was the most common myometrial lesion seen (Table 1).

**Table 1:** Spectrum of lesions encountered in the frozen section.

Spectrum of lesions encountered in the frozen section	n	%
Benign endocervical polyp	1	1.3%
Carcinosarcoma	1	1.3%
Leiomyosarcoma	1	1.3%
Atypical endometrial hyperplasia	2	2.6%
Endometrial stromal sarcoma	2	2.6%
Benign endometrial polyp	8	10.5%
Leiomyoma with benign endometrial polyp	10	13.1%
Endometrial carcinoma	11	14.4%
Leiomyoma with degeneration	16	21%
Leiomyoma	24	31.5%

**Figure 6:**
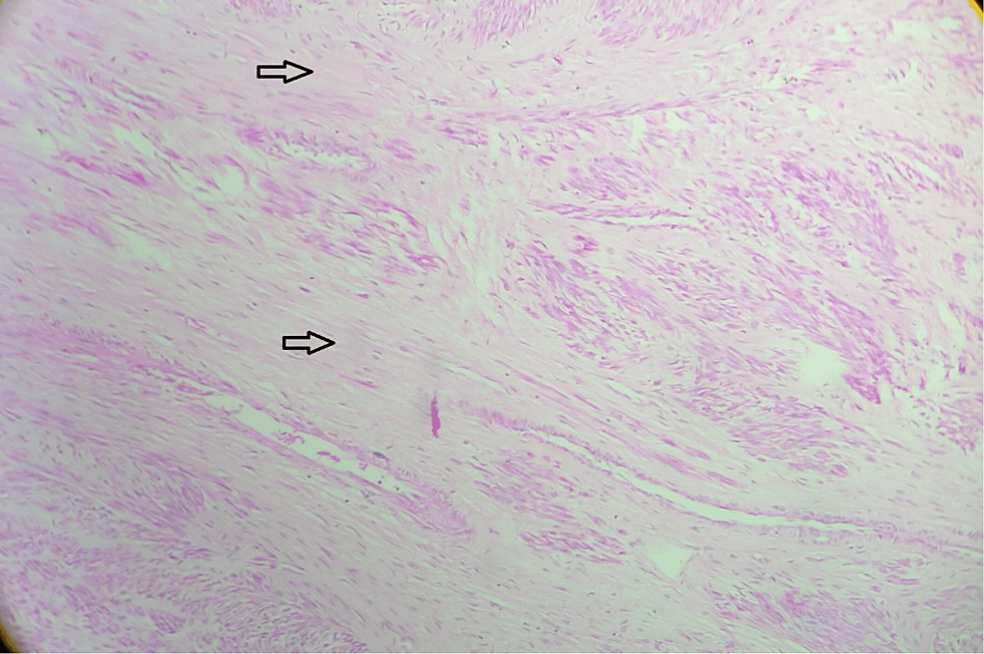
Hyaline degeneration of leiomyoma; hematoxylin and eosin stain; scanner view. The section shows hyaline degenerative changes seen as homogenous eosinophilic changes in the smooth muscle cells of the leiomyoma (indicated by both the arrows in the figure). Source: Neelayadakshi B.

Of the 50 cases (65.6%) of leiomyoma encountered, 16 cases (32%) had extensive degenerative changes. The most common degeneration seen in the fibroid was hyaline degeneration (50%) (Figure 6).

**Figure 7:**
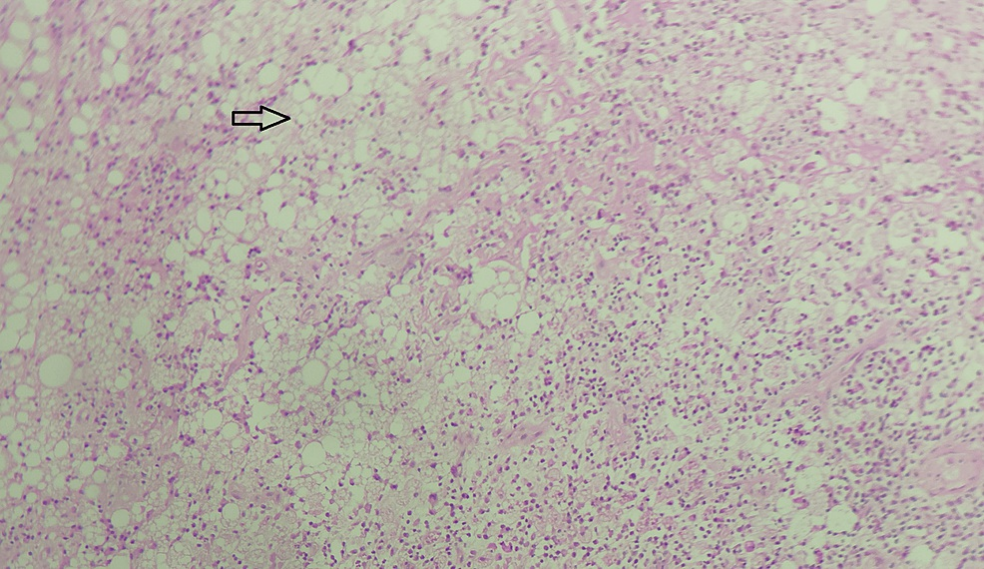
Xanthogranulomatous degeneration of leiomyoma; x10 magnification; hematoxylin and eosin stain. Myometrium shows extensive xanthogranulomatous reaction composed of extensive foamy macrophages (indicated by the arrow), lymphoplasmacytic cell infiltrate, and sheets of neutrophils in focal areas amidst intervening smooth muscles. No atypia or malignancy was noted. Source: Neelayadakshi B.

The least common was xanthogranulomatous degeneration (6.25%) (Figure 7; Table 2).

**Table 2:** Types of degenerations encountered in leiomyoma.

Types of degenerations encountered in leiomyoma	n	%
Hyaline degeneration	8	50%
Cystic degeneration	5	31.2%
Myxoid degeneration	2	12.5%
Xanthogranulomatous degeneration	1	6.25%

The mean age of the women for whom frozen section analysis was sent in the present study was 49.2 years. The concordance between frozen section findings and the subsequent histopathological diagnosis in our study was found to be 98.7% (Table 3).

**Table 3:** Concordance between frozen section and routine HPE diagnosis. HPE, histopathological examination

Concordance	n	%
Concordance	75	98.7%
No concordance	1	1.31%
Total	76	100%

In only one case, it was diagnosed as leiomyoma in frozen section analysis, which turned out to be low-grade endometrial stromal sarcoma in the subsequent HPE (1, 1.31% nonconcordance; Table 3). The diagnostic value of the frozen section, according to the status of malignancy in this study, was found to be as follows: sensitivity, 94.11%; specificity, 100%; and accuracy, 98.6% (Table 4).

**Table 4:** Diagnostic value of the frozen section, according to the status of malignancy in this study.

Statistical value	Percentage
Sensitivity	94.11%
Specificity	100%
Accuracy	98.6%

## Discussion

Frozen section analysis in uterine pathology is indicated to ensure that adequate tissue has been sampled for diagnosis, to determine the nature of the uterine lesion - whether benign or malignant, to assess the margins and to determine if the tumor has spread or not, and thus to plan the extent of surgery in the operation theatre [3]. Intra-operative frozen section can be very useful in identifying patients who have a high risk for extrauterine spread and those who may require lymphadenectomy [3].

In the present study, out of the total 16 cases (21.05%) that were reported as malignant in frozen section analysis, all 16 (21.05%) turned out to be malignant in the subsequent histopathological report, thus showing 100% concordance for malignancy in frozen section analysis. Out of the 60 cases (78.9%) reported to be benign in frozen section analysis, 59 (77.6%) turned out to be benign, while one case (1.31%) turned out to be malignant, thus showing 98.7% concordance (Table 5).

**Table 5:** Correlation of frozen section diagnosis with subsequent routine HPE diagnosis. HPE, histopathological examination

		Routine HPE diagnosis		
		Benign	Malignant	Total
Frozen section analysis	Benign	59 (77.6%)	1 (1.31%)	60 (78.9%)
	Malignant	0	16 (21.05%)	16 (21.05%)
	Total	59 (77.6%)	17 (22.36%)	

**Figure 8:**
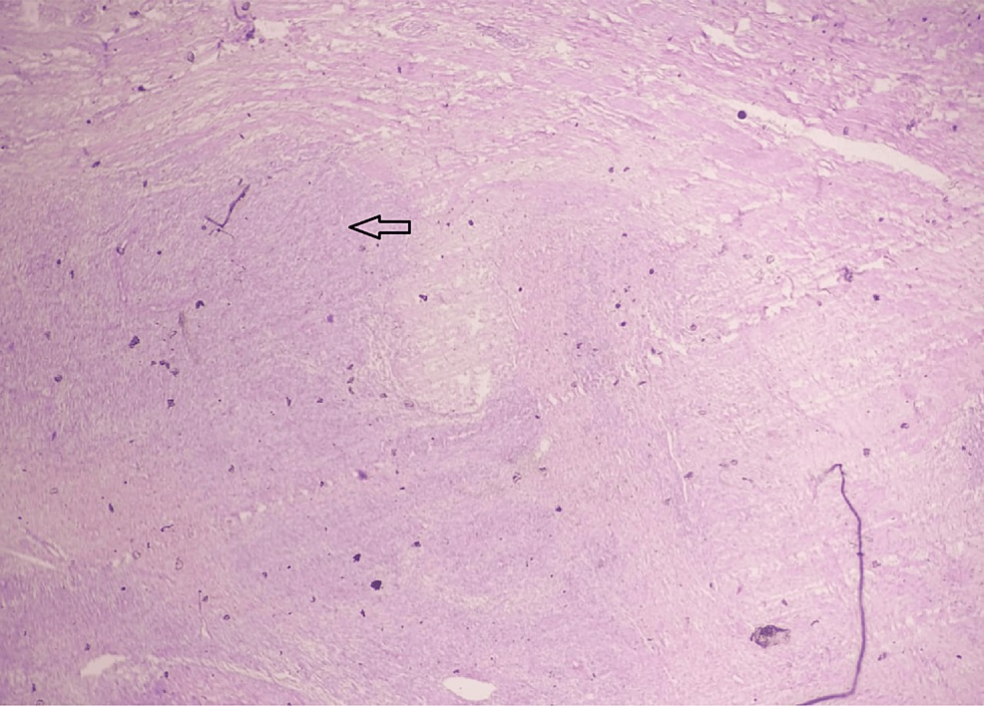
Low-grade endometrial stromal sarcoma, the HPE of which was nonconcordant with the frozen section; HPE image, hematoxylin and eosin stain; scanner view. The section shows tongue-like projections of the low-grade endometrial sarcoma seen invading the adjacent myometrium (indicated by the arrow), which was missed in the frozen section analysis due to sampling error. Source: Neelayadakshi B.

The one case that was not concordant was reported as leiomyoma in frozen section analysis, while it turned out to be low-grade endometrial stromal sarcoma in the subsequent HPE (Figure 8). The nonconcordance was due to sampling error during frozen section analysis.

In our study, the predominant age group of women for whom the frozen section analysis was sent was 41-50 years, with a mean age of 49.2 years. The spectrum of malignant mesenchymal tumors encountered in our study was low-grade endometrial stromal sarcoma, high-grade endometrial stromal sarcoma, leiomyosarcoma, and carcinosarcoma (malignant mixed mullerian tumor). Other rare cases encountered in our study included xanthogranulomatous degeneration of leiomyoma.

The most common malignancy seen in the female genital tract worldwide is endometrial carcinoma [1,4,5]. In our study as well, the most commonly reported cancer sent for frozen section analysis was endometrial carcinoma (14.4% overall) (Figure 1).

The mean age of the women for whom frozen section analysis was sent in this study was 49.2 years. This was similar to the observations noted in the study done by Aidos et al., in which the mean age noted was 51 years [4]. However, in the study done by Stephan et al., the mean age of women included in the study was 60.1 years [1]. According to this study, the frozen section's sensitivity is 94.11%, which was almost similar to the study done by Aidos et al., where the sensitivity was 96.9% [4]. However, the sensitivity of the frozen section in the studies done by Wang et al. and Stephan et al. was 84.2% and 90%, respectively [2,1]. The specificity of the frozen section according to this study was found to be high (100%), which correlated with the findings of the studies conducted by Aidos et al., Wang et al., and Stephan et al., in which the specificity was found to be 100%, 97.3%, and 100%, respectively [4,2,1]. The accuracy of the frozen section, as per this study, was also found to be high (98.6%), similar to observations in other studies by Stephan et al., Aidos et al., and Wang et al., where accuracies were found to be 97.5%, 93.75%, and 92.9%, respectively [1,4,2]. This demonstrates that frozen section analysis is a valuable tool for surgeons in on-table decision-making regarding the management of uterine masses during surgery (Table 6).

**Table 6:** Comparison between various studies.

Study variables	Present study	Wang et al.	Stephan et al.	Aidos et al.
Type of study	Hospital-based study	Hospital-based study	Hospital-based study	Hospital-based study
Number of cases	76	56	116	102
Sensitivity	94.11%	84.2%	90%	96.9%
Specificity	100%	97.3%	100%	100%
Accuracy	98.6%	92.9%	97.5%	93.75%
Mean age	49.2 years	-	60.1 years	51 years

Xanthogranulomatous inflammation is a rare form of chronic inflammation, which destroys the organ tissue that is affected [6,7,8]. It is most commonly seen in the kidneys and the gall bladder [6,9]. However, it is rarely seen in the female genital tract [6]. In the female genital tract, it has been reported involving the endometrium [6,10,11], fallopian tubes [6,12,13], and ovary [6,14], primarily presenting as endometritis and/or salpingitis, with a tubo-ovarian abscess [8]. However, xanthogranulomatous inflammation with uterine myometritis, without endometritis has rarely been reported in the literature [8].

In xanthogranulomatous inflammation, there is the presence of many lipid-laden macrophages seen in a background of lymphocytes and plasma cells [6]. Occasionally multinucleated giant cells may also be seen, and fibrosis may also be a feature [6]. In our case, there was the presence of foamy macrophages, lymphoplasmacytic cell infiltrate, and sheets of neutrophils, amidst intervening smooth muscle cells. However, no multinucleated giant cells or fibrosis was seen in our case.

**Figure 9:**
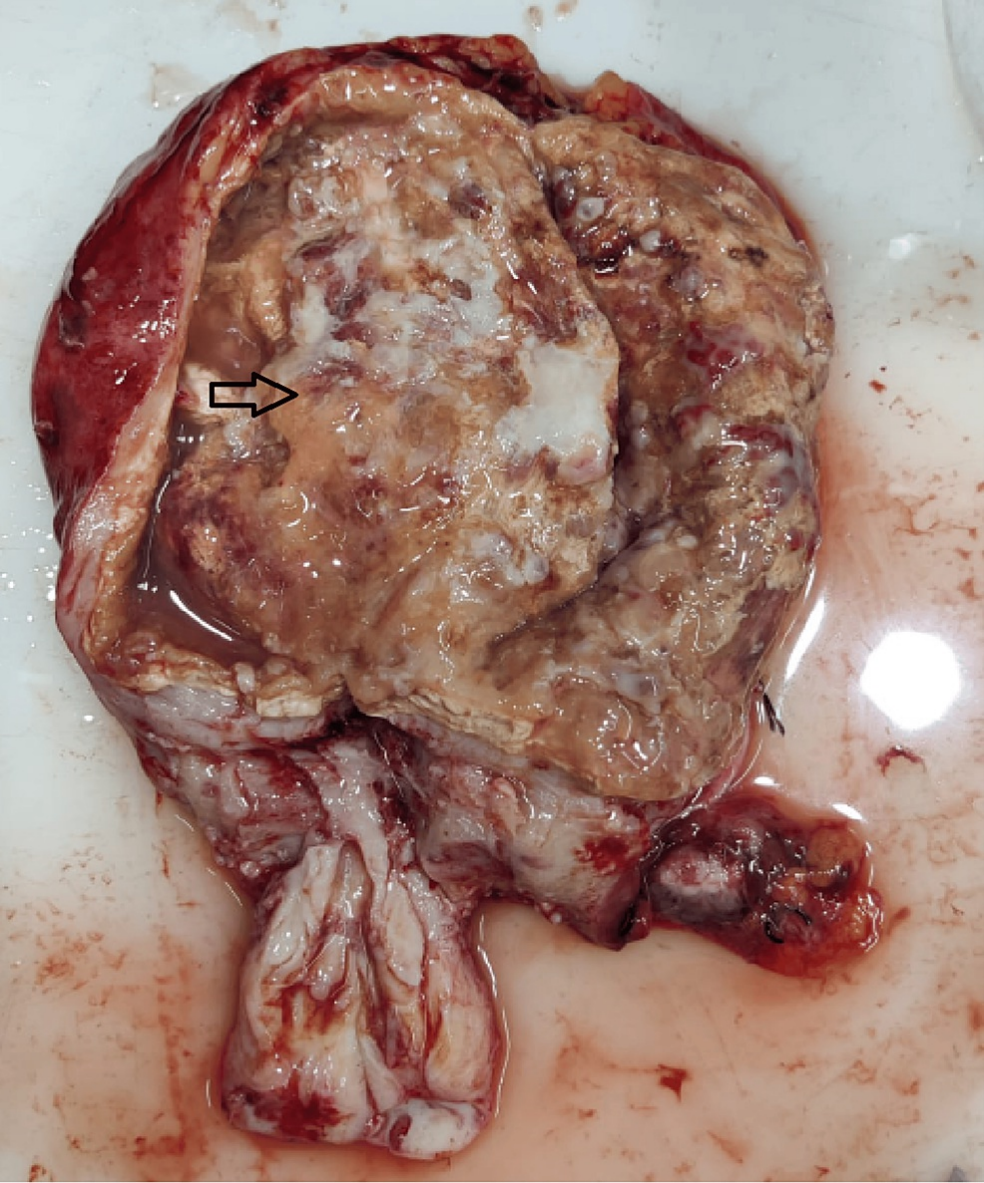
Xanthogranulomatous degeneration of leiomyoma in the fundus of the uterus - Gross. Uterus with cervix measuring 15 cm x 11 cm x 5 cm. Endometrial thickness could not be made out. Cut surface showed a lesion in the fundus measuring 8.5 cm in diameter (indicated by the arrow); purulent material was let out from the lesion. Source: Neelayadakshi B.

**Figure 10:**
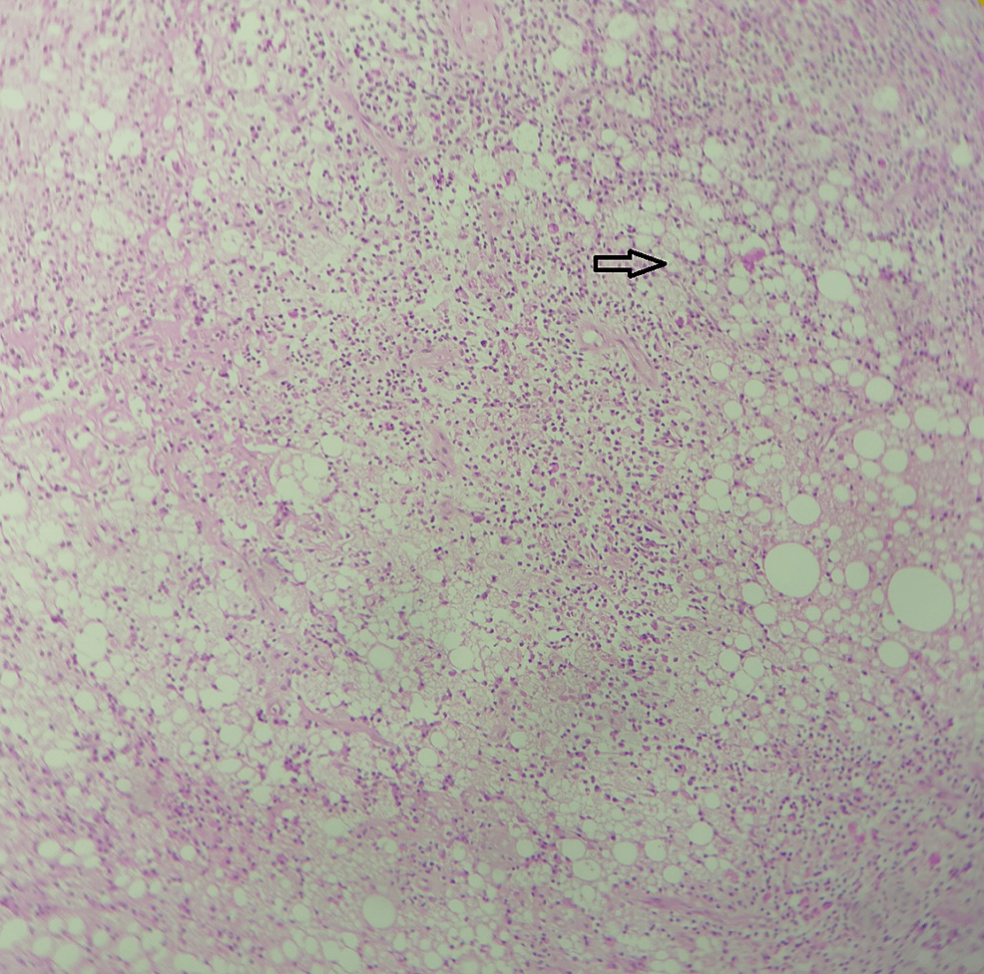
Xanthogranulomatous degeneration of leiomyoma in the uterus - microscopic picture x10 magnification; hematoxylin and eosin stain. Myometrium shows a xanthogranulomatous reaction composed of extensive foamy macrophages (indicated by the arrow), lymphoplasmacytic cell infiltrate, and sheets of neutrophils in focal areas amidst intervening smooth muscles. No atypia or malignancy was noted. Source: Neelayadakshi B.

The most common tumor affecting the uterine corpus is leiomyoma [6], as seen in our study as well, where the most commonly detected uterine mass in the frozen section was leiomyoma with or without degeneration (65.6% overall). When these tumors grow big, they undergo degeneration due to decreased blood supply [6]. Different types of degeneration have been reported in leiomyoma, which includes hyaline degeneration, myxoid degeneration, and red degeneration [6]. However, xanthogranulomatous degeneration involving leiomyoma is a very rare entity, and only a few cases have been reported so far in the literature [6]. In our case, the patient was a 57-year-old postmenopausal woman who presented to the gynecology outpatient department with complaints of abdomen pain and abdominal distension on and off for 10 years. There was an increase in severity for about two weeks. Frozen section analysis was conducted, and the frozen section diagnosis was given as degenerative change in the fibroid. Subsequent histopathological examination showed myometrium having xanthogranulomatous reaction composed of numerous foamy macrophages, lymphoplasmacytic infiltrate, and fibrinoid necrosis, along with sheets of neutrophils in focal areas amidst intervening smooth muscles. The histopathological examination findings were given as xanthogranulomatous degeneration of leiomyoma (Figures 9-10).

## Conclusions

The intraoperative frozen section analysis is an extremely important diagnostic tool for surgeons. However, we should be aware of its limitations such as the quality of the sections is generally inferior to the sections in the histopathological examination, there can be sampling error, the representative areas may be missed, and there can be more artifacts seen under the frozen section. This study confirms the diagnostic value of frozen section analysis in the evaluation and management of uterine tumors in our hospital. The accuracy, sensitivity, and specificity rates for frozen section analysis were high in our study. This shows that frozen section diagnoses can be very valuable in the clinical management of uterine tumors. Careful gross examination along with sampling from representative areas and good communication between the reporting pathologist and operating surgeon may help in avoiding its limitations.
